# A New Strategy for Efficient Screening and Identification of Monoclonal Antibodies against Oncogenic Avian Herpesvirus Utilizing CRISPR/Cas9-Based Gene-Editing Technology

**DOI:** 10.3390/v14092045

**Published:** 2022-09-14

**Authors:** Man Teng, Zi-Yu Zhou, Yongxiu Yao, Venugopal Nair, Gai-Ping Zhang, Jun Luo

**Affiliations:** 1 Key Laboratory of Animal Immunology, Ministry of Agriculture and Rural Affairs of China & Henan Provincial Key Laboratory of Animal Immunology, Henan Academy of Agricultural Sciences, Zhengzhou 450002, China; 2 UK-China Centre of Excellence for Research on Avian Diseases, Henan Academy of Agricultural Sciences, Zhengzhou 450002, China; 3 College of Animal Science and Technology, Henan University of Science and Technology, Luoyang 471003, China; 4 The Pirbright Institute & UK-China Centre of Excellence for Research on Avian Diseases, Pirbright, Ash Road, Guildford GU24 0NF, UK; 5 International Joint Research Center of National Animal Immunology & College of Veterinary Medicine, Henan Agricultural University, Zhengzhou 450002, China; 6 Jiangsu Co-Innovation Center for Prevention and Control of Important Animal Infectious Disease and Zoonoses, Yangzhou University, Yangzhou 225009, China

**Keywords:** herpesvirus, MDV, monoclonal antibody, CRISPR/Cas9, pp38, IFA, Western blot, confocal microscopy analysis

## Abstract

Marek’s disease virus (MDV) is an important oncogenic α-herpesvirus that induces Marek’s disease (MD), characterized by severe immunosuppression and rapid-onset T-cell lymphomas in its natural chicken hosts. Historically, MD is regarded as an ideal biomedical model for studying virally induced cancers. Monoclonal antibodies (mAbs) against viral or host antigenic epitopes are crucial for virology research, especially in the exploration of gene functions, clinical therapy, and the development of diagnostic reagents. Utilizing the CRISPR/Cas9-based gene-editing technology, we produced a pp38-deleted MDV-1 mutant—GX0101Δpp38—and used it for the rapid screening and identification of pp38-specific mAbs from a pool of MDV-specific antibodies from 34 hybridomas. The cross-staining of parental and mutated MDV plaques with hybridoma supernatants was first performed by immunofluorescence assay (IFA). Four monoclonal hybridomas—namely, 4F9, 31G7, 34F2, and 35G9—were demonstrated to secrete specific antibodies against MDV-1’s pp38 protein, which was further confirmed by IFA staining and confocal analysis. Further experiments using Western blotting, immunoprecipitation (IP), liquid chromatography–tandem mass spectrometry (LC–MS/MS), and immunohistochemistry (IHC) analysis demonstrated that the pp38-specific mAb 31G7 has high specificity and wide application potential for further research in MD biology. To the best of our knowledge, this is the first demonstration of the use of CRISPR/Cas9-based gene-editing technology for efficient screening and identification of mAbs against a specific viral protein, and provides a meaningful reference for the future production of antibodies against other viruses—especially for large DNA viruses such as herpesviruses.

## 1. Introduction

Marek’s disease (MD) is one of the most important immunosuppressive and neoplastic diseases, caused by the infection of natural chicken hosts with pathogenic/oncogenic serotype 1 Marek’s disease virus (MDV-1). MD is estimated to cause annual direct economic losses of more than USD 1 billion to the poultry industry worldwide [1,2]. As an important oncogenic herpesvirus, MDV-1 belongs to the subfamily Alphaherpesvirinae of Herpesviridae, and has been recently reclassified as *Gallid* *alphaherpesvirus* 2 (GaAHV-2) [3]. It can establish and maintain latent infection, and then induce rapid-onset T-cell lymphomas in multiple visceral organs of virus-infected birds. Interestingly, the lymphomas induced by MDV-1 infection can be prevented by MD vaccine immunization, so MDV-1 has been historically regarded as an excellent biomedical model for the study of virus-induced cancers [4]. In recent years, it has been shown that under the long duration and high immune pressure of universal MD vaccination, continuous increases in virulence and related genovariation of epidemic MDV strains have occurred, and the threat of MD outbreaks to the global poultry industry has never been eliminated [5,6].

Undoubtedly, elucidation of the mechanisms underlying MDV’s pathogenesis and/or oncogenesis is critical for the efficient control of MD. However, among hundreds of MDV-encoded protein-coding and non-coding RNA genes, most of their potential roles and functions involved in the virus’ life cycle and the development of MD tumors remain unclear. Seven MDV-1-specific genes—including the MDV EcoRI-Q (meq), 38 kD phosphorylated protein (pp38), virus-encoded telomerase RNA (vTR), viral lipase homologue (vLIP), viral IL-8 (vIL8), 1.8 kb mRNA, and the latency-associated transcripts (LATs)—have been directly linked to pathogenesis [7]. Presently, the meq gene encoding a basic leucine zipper protein has been characterized as a major oncogene responsible for MDV’s tumorigenesis, although the other genes may also play important roles in triggering the development of MD tumors [8]. For a long time, the molecular mechanism of MD’s tumorigenesis has been an attractive research focus for many virologists, and the development of monoclonal antibodies (mAbs) against important viral proteins is crucial for studies on MDV’s pathogenesis/oncogenesis, diagnosis techniques, and reagents. In previous studies [9,10,11], a series of mAbs against all three serotypes of MDV—including MDV-1, MDV-2 (*Gallid* *alphaherpesvirus* 3 (GaAHV-3)), and MDV-3/HVT (the turkey herpesvirus, *Meleagrid* *alphaherpesvirus* 1 (MeAHV-1))—were developed and prepared in the 1980s–1990s. However, due to the technique limitations at that time, the amounts, identities, and potential usages of these mAbs were either insufficient or had not been well defined, and specific mAbs were developed only for a limited number of viral proteins to support research on MD’s biology. With the great progresses in biological technology and virology research in recent years, the development and preparation of more mAbs with high specificity against MDV proteins is obviously necessary and urgent.

Recently, the new generation of gene-editing technology based on the CRISPR/Cas9 system has been well developed and widely applied in several aspects of virology research [12]. Due to its advantages of simplicity, quickness, and high efficiency, the CRISPR/Cas9-based gene-editing technology has been successfully applied to operate and reconstitute the viral genomes of large DNA viruses—especially the family of herpesviruses, including MDV. Since the first report in 2016 [13], the CRISPR/Cas9 system applied to edit the lytic or integrated MDV genomes has been successfully established in both virus-infected chicken embryo fibroblast (CEF) cells and virally transformed lymphoblastoid cell lines, achieving important advances in the functional studies of both protein-coding and non-coding RNA genes, as well as the development of innovative MD vaccines [14,15,16,17,18,19,20]. In this report, we used an immunofluorescence assay (IFA) to stain the viral plaques produced in CEFs by infection with a very virulent (vv) MDV strain (GX0101) and its mutant lacking the phosphorylated protein 38 (pp38) gene, generated through the CRISPR/Cas9-based gene-editing system, for screening of a pool of mAbs against MDV proteins. Additional experimental data from IFA staining, Western blot analysis, immunoprecipitation (IP), mass spectrometry (MS), and immunohistochemistry (IHC) convincingly confirmed that the new strategy for identifying mAbs against MDV-specific proteins, supported by CRISPR/Cas9-based targeted gene editing, is a powerful method for antibody discovery. To the best of our knowledge, this is the first demonstration of yet another application of CRISPR/Cas9-based gene-editing technology for quick screening and identification of mAbs against a viral protein, providing an important reference for the future production of specific mAbs for other viruses—especially for those of the large DNA viruses, such as herpesviruses.

## 2. Materials and Methods

### 2.1. Ethics Statement

All experimental protocols were approved by the Laboratory Animal Management Committee of the Key Laboratory of Animal Immunology, Ministry of Agriculture and Rural Affairs, People’s Republic of China. Animal experiments were conducted following the protocols of the Laboratory Animal Guidelines for Ethical Review of Animal Welfare, permitted by the State Administration for Market Regulation and Standardization Administration of China (permit no. GB/T 35892–2018).

### 2.2. Viruses and Cells

The vvMDV strain GX0101 was used as a parental virus for the generation of a pp38-deleted MDV mutant, together with the other five virulent or vaccine strains Md5, GA, CVI988 (representing other MDV-1 strains), HVT (representing MDV-3 strains), and SB-1 (representing MDV-2 strains), for the detection of the reaction spectrum of mAbs. Primary CEF monolayers prepared from 9-day-old specific-pathogen-free (SPF) embryos (Beijing Boehringer Ingelheim Vital Biotechnology Co., Ltd., China) were maintained in M199 medium (Gibco, USA) supplemented with 5% fetal bovine serum (FBS, Sigma, USA), 100 units/mL penicillin, 100 µg/mL streptomycin, and 10% tryptose phosphate broth (TPB) (Solarbio, China). The 293T cells were maintained in Dulbecco’s modified Eagle medium (DMEM) (Gibco, USA) supplemented with 10% FBS and the same penicillin–streptomycin. All of the cells and virus cultures were incubated at 38.5 °C in a 5% CO_2_ incubator.

### 2.3. sgRNA Plasmid Construct

The specific guide RNAs (gRNAs) targeting the MDV-1 pp38 gene of the GX0101 virus were designed using the online software GenCRISPR gRNA Design Tool (GenScript, USA). Each of three gRNAs targeting the upstream or downstream of the pp38 gene, as listed in Table 1, were synthesized (Sangon Biotech, Shanghai, China) and cloned into the BbsI restriction enzyme sites of pX459v2.0 to construct the Cas9/gRNA expression plasmids pX459-gRF1~pX459-gRF3 and pX459-gRR1~pX459-gRR3, respectively. The PCR primers used for amplification and identification of the MDV-1 pp38 gene are listed in Table 1.

### 2.4. Generation of a pp38-Edited GX0101 Virus

The CEF monolayers in 24-well plates were first co-transfected with different pairs of plasmids pX459-gRF1~pX459-gRF3 and pX459-gRR1~pX459-gRR3 (Table 1) using the Trans IT-X2™ Dynamic Delivery System (Mirus Bio, USA) according to the manufacturer’s instructions. At 24 h post-transfection (hpt), the cells were infected with GX0101 at 0.01 PFU/cell. The transfected/infected CEFs were harvested at 48 hpt, and then lyzed in 1× squishing buffer (10 mM Tris-HCl, pH 8.0, 1 mM EDTA, 25 mM NaCl, and 200 µg/mL proteinase K) at 65 °C for 30 min, followed by 95 °C for 5 min. PCR amplification was performed to analyze the gene-editing efficiency of gRNA combinations using pp38-specific primers (Table 1). The smaller bands of mutated PCR products were purified and sequenced to confirm the gRNA-mediated specific mutagenesis. Then, fresh CEF monolayers in 6-well plates were infected with the primarily mutated GX0101/CEF cultures via a limited dilution method to produce individual MDV plaques. Once the plaques were visible at 3–5 days post-infection (pi), the individual plaques were picked up into CEF monolayers in 24-well plates for propagation, and were detected by PCR analysis again. For the purification of the pp38-mutated virus strain, two rounds of MDV plaque cloning, purification, PCR analysis, and DNA sequencing were performed until the PCR products from all of the individual plaques contained only the mutated smaller bands. Finally, the purified MDV mutant was passaged 15 times and frozen in liquid nitrogen for further experiments.

### 2.5. Immunofluorescence Assay (IFA)

For characterization of the pp38-deleted MDV mutant, the expression of the MDV-1-specific proteins MEQ and pp38 in the parental and mutant GX0101 virus-infected CEFs was detected by immunofluorescence assay (IFA), as previously described [17]. The virus-infected cells were first incubated with the rabbit anti-MEQ polyclonal antibody and mouse anti-pp38 mAb BD1 (both produced at the Pirbright Institute), and then incubated with the secondary antibodies DyLight 594 Goat Anti-Rabbit IgG and DyLight 488 Goat Anti-Mouse IgG (Abbkine, USA), respectively. Images were taken using an inverted fluorescence microscope and a confocal microscope (Zeiss, Germany).

### 2.6. Reverse-Transcription Quantitative PCR (RT-qPCR)

The relative expression levels of the MDV-1 genes pp38, glycoprotein B (gB), repeat long open reading frame 6 (RLORF6), unique long open reading frame 6 (UL6), UL13, UL42, and UL52 in virus-infected CEF cells were determined by RT-qPCR in comparison to the transcripts of the viral oncogene meq, as previously described [14]. The total cellular RNA was extracted from virus-infected CEFs using TRIzol reagent (Invitrogen, USA), and 1 µg of RNA was reverse-transcribed using 5×PrimeScript RT Master (TaKaRa, Japan) according to the manufacturer’s protocols. The 7500 Fast Real-Time PCR Systems (Applied Biosystems, Life Technologies, USA) was used for qPCR amplification with FastStart Universal SYBR^®^ Green Master (Roche, Switzerland). The details of primers specific to the MDV-1 viral genes are listed in Table 1. The experiments were repeated independently in triplicate. The data were finally calculated with the 2^−ΔΔCt^ method, and the relative expression levels were calculated as fold changes compared to those from parental GX0101 virus-infected cells.

### 2.7. Construction of a Pool of mAbs against MDV Proteins

The cytoplasmic and nuclear proteins were separately extracted and purified from GX0101-infected CEF cells using the NE-PER^®^ Nuclear and Cytoplasmic Extraction Reagents (Thermo Fisher Scientific, USA), and then were equally mixed and used as immunogens (30 μg/mouse in Freund’s adjuvant) to immunize 6-week-old female BALB/C mice three times with 3-week intervals, followed by a final immunization with the same dose of purified cellular protein 3 days before cell fusion. Conventional cell fusion technology was employed to generate hybridomas. The supernatants of either monoclonal or polyclonal hybridomas were screened for positive antibodies against MDV plaques by IFA staining, as described above. The positive supernatants were collected for further experiments, and the hybridoma cell strains were stored in liquid nitrogen to provide a pool of mAbs against MDV proteins.

### 2.8. Cross-Screening of pp38-Specific mAbs

The CEF monolayers in 24-well plates were separately infected with GX0101 and its pp38-deleted mutant to produce MDV plaques, and then were fixed and incubated with positive supernatants from the pool of MDV-specific mAbs to perform IFA staining, as described above. The ones showing a specific positive reaction to GX0101 plaques, but not to the pp38-deleted MDV mutant virus, were cross-screened and picked up through observation under fluorescence microscopy, and were preliminarily regarded as positive mAb candidates specific to MDV-1 pp38 proteins.

### 2.9. Protein Expression and Examination of mAb Specificity

The full-length sequence of MDV-1’s pp38 gene was synthesized and cloned into the eukaryotic expression plasmid pEGFP-N1 (SunYa, China) to generate the pEGFP-N1-pp38 plasmid, followed by transfection into 293T cells in 24-well plates using LipofectamineTM 2000 (Thermo Fisher Scientific, Basingstoke, UK) according to the manufacturer’s instructions. Two days later, the cells were fixed with precooled methanol/acetone (*v*/*v* = 1:1) and blocked with 5% skimmed milk, and then incubated with the positive supernatants of pp38 mAb candidates, followed by incubation with the secondary antibody DyLight 594 Goat Anti-Mouse IgG (Abbkine, USA). The pp38-specific mAb BD1 served as a positive control. Finally, the results were checked and recorded under a fluorescence microscope.

### 2.10. Confocal Microscopy Analysis

The confocal microscopy analysis was performed to observe the cellular localization of the specific viral proteins recognized by the mAbs in MDV-infected CEFs, similarly to the IFA staining described above. The anti-pp38 mAbs (4F9, 31G7, 34F2, or 35G9) and anti-gB mAb HB3 (produced at The Pirbright Institute, UK) were used as primary antibodies, followed by incubation with the corresponding secondary antibodies DyLight 594 Goat Anti-Rabbit IgG and DyLight 488 Goat Anti-Mouse IgG (Abbkine, USA), respectively. Images were taken using a confocal microscope (Zeiss, Germany).

### 2.11. Isotype Characterization of pp38 mAbs

The hybridomas secreting pp38-specfic mAbs with stronger reactivity and higher titers were further cloned by limited dilution to establish the monoclonality of the hybridoma cell strains. The ascitic fluids were generated through intraperitoneal injection of positive monoclonal hybridomas into BALB/C mice, and the titers were determined by IFA staining as described above. The isotypes of the mAbs were determined using the Mouse Monoclonal Antibody Isotyping Kit (Proteintech, USA).

### 2.12. Reaction Spectrum of pp38 mAbs to MDVs

To investigate the reaction specificities of pp38 mAbs to distinct serotypes of MDV, the CEF monolayers were separately infected with Md5, GX0101, GA, CVI988, HVT, and SB-1 viruses to produce MDV plaques, and then the CEF cells were fixed and incubated with the newly identified pp38 mAb 31G7 to perform IFA staining. Simultaneously, the virus-infected CEF cells were collected and boiled with 1×SDS–PAGE Sample Loading Buffer (Beyotime, China) for 10 min. The samples were separated on Bolt^TM^ Bis-Tris Plus 4%–12% precast gel (Invitrogen), and the resolved proteins were then transferred onto PVDF membranes by iBlot^®^ 2 PVDF Regular Stacks (Invitrogen, USA). The expression levels of pp38 proteins in Md5, GX0101, GA, CVI988, HVT, and SB-1-infected CEFs were determined by incubating mAb 31G7 and HRP-labeled Goat Anti-Mouse IgG (Pharmacia, USA) sequentially, and were finally visualized using NcmECL Ultra (NCM Biotech, China). In all cases, chicken β-actin (Sangon Biotech, Shanghai, China) served as the loading control.

### 2.13. Immunoprecipitation (IP)

The GX0101-infected CEF cultures in T75 flasks were collected and washed twice with precooled phosphate-buffered saline (PBS). The cells were lyzed with 1 mL of cell lysis buffer (containing 1 mM of PMSF) on ice for 30 min, and were centrifuged at 12,000 rpm at 4 °C for 15 min to collect supernatant, which was further incubated with 50 µL of protein A/g magnetic beads (MedChemExpress, China) adsorbed with pp38-specific mAb 31G7. As a negative control, the uninfected CEF cells and the negative control antibody (NCAb) anti-JEV-E (produced at the Key Laboratory of Animal Immunology, HAAS, China) were used. The magnetic beads were separated and washed with Tween-20/PBS. After the addition of 30 µL of 1×SDS–PAGE loading buffer, the lysate was boiled for 5 min as preparation for SDS–PAGE electrophoresis. The gel was dyed with the Fast Silver Stain Kit (Beyotime, China) and the target bands were separated by their correct molecular weight, and then sent to OE Biotech (Shanghai, China) for liquid chromatography–tandem mass spectrometry (LC–MS/MS) analysis.

### 2.14. Immunohistochemistry (IHC)

The feather follicle samples (provided by Dr. Aijun Sun, Henan Agricultural University, China) were randomly collected during the early infection stage from Md5-challenged or mock-infected birds, and were immersed in O.C.T., immediately frozen in liquid nitrogen, and stored at −80 °C until use. Then, the tissue sections were prepared and subjected to immunostaining as described previously [21], using MDV-1 pp38 specific mAb 31G7 and the DAB Kit (Cowin Biosciences, China).

## 3. Results

### 3.1. Generation of a pp38-Deleted MDV Mutant Using the CRISPR/Cas9 System

**Figure 1:**
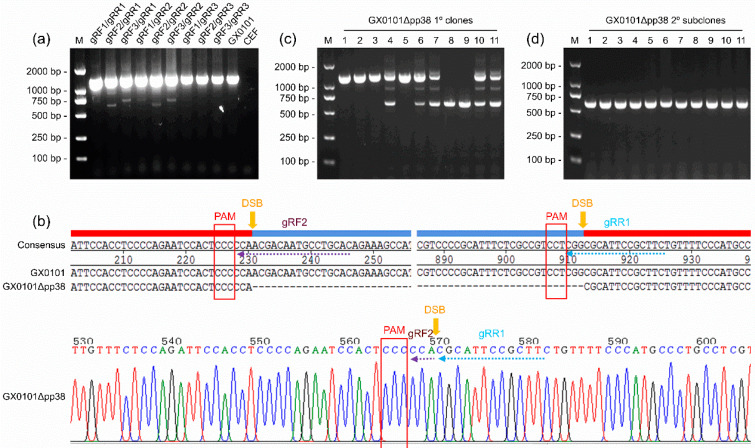
PCR analysis and sequence alignment of the double-strand breaks in pp38-deleted MDV mutants: (**a**) PCR analysis of the mutated or wild-type pp38 genes of GX0101 edited by crossed combinations of different gRNAs. (**b**) Sequence alignment of double-strand breaks (DSBs) in pp38 genes mutated by the gRNA combination gRF2/gRR1. (**c**,**d**) PCR analysis of pp38 deletions in single MDV plaques from the first and second rounds of virus purification, respectively. Due to limited space, only some of the results are shown here. The entire or broken gRNA sequences and protospacer-adjacent motifs (PAMs) are shown by same-colored arrows or square frames in red, respectively.

For the generation of a pp38-deleted MDV mutant virus, we designed six gRNAs targeting the MDV-1-specific pp38 gene and cloned into pX459v2.0 to generate the Cas9/gRNA expression plasmids pX459-gRF1~pX459-gRF3 and pX459-gRR1~pX459-gRR3 (Table 2). The different pairs of pX459-gRF/gRR plasmid combinations were co-transfected into CEFs and infected with the vvMDV strain of the GX0101 virus. The efficacy of pp38 gene editing was analyzed by PCR, and the results showed that in the GX0101-infected CEFs, most of the crossed gRNA combinations worked efficiently. As demonstrated in Figure 1a, a series of smaller mutated bands—about 600 bp in length—were observed in PCR products amplified from the virus-infected CEFs that had been co-transfected with six pX459-gRF/gRR pairs, i.e., gRF1/gRR1, gRF2/gRR1, gRF3/gRR1, gRF1/gRR2, gRF2/gRR2, and gRF3/gRR2. In the other groups of gRNA co-transfected, untransfected, or uninfected mock CEF cells, only the wild-type bands of PCR products with a length of 1295 bp were observed. After gene cloning and DNA sequencing to confirm the successful mutagenesis of the pp38 gene mediated by the gRF2/gRR1 combination (Figure 1b), the CEF cells containing mutated GX0101 viruses were transferred to fresh CEF monolayers to produce single plaques. In total, 72 single viral plaques were picked up for the first round of purification of pp38-deleted mutants, of which the positive rate of purified single viral plaques containing only smaller mutated bands was 5.6% (4/72) (Figure 1c). The primarily purified plaque was passaged and cloned again for the second round of purification, and the final PCR analysis showed that the products amplified from all of the detected single viral plaques contained only one smaller band, with a positive mutation rate of 100.0% (Figure 1d). Thus, the pp38-deleted MDV mutant—namely, GX0101∆pp38—was finally passaged and expanded to make virus stocks for subsequent experiments.

### 3.2. Identification of the pp38-Deleted MDV Mutant GX0101∆pp38

**Figure 2:**
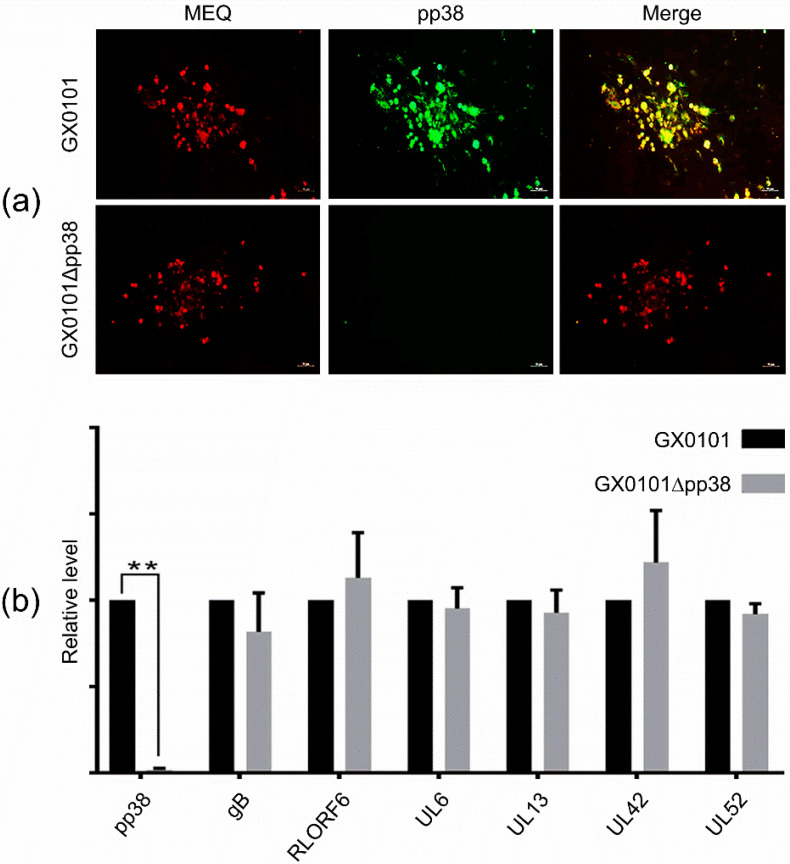
IFA staining and RT-qPCR analysis of the expression of viral genes in MDV-infected CEFs: (**a**) Expression of the MEQ and pp38 proteins in GX0101 or GX0101∆pp38-infected CEFs, as detected by immunofluorescence assay (scale bar = 50 µm). (**b**) Relative expression levels of MDV-1 genes in GX0101- or GX0101∆pp38-infected CEFs, as determined by RT-qPCR analysis. All of the experiments were independently repeated three times, and the gene expression levels were normalized to that of the MDV oncogene meq. Asterisks (*) indicate statistically significant differences between the pp38-deleted mutant and the parental GX0101; **, *p* < 0.01.

The deletion of the MDV-1 pp38 gene from the GX0101 virus was further confirmed by IFA staining. As demonstrated in Figure 2a, the MEQ protein was expressed in both parental GX0101 and mutant GX0101∆pp38-infected CEF cells, as expected, while the pp38 protein was only expressed in the parental GX0101-infected cells—not in the GX0101∆pp38-infected cells. In addition, in order to evaluate whether the deletion of the pp38 gene affects expression of the other viral protein-coding genes encoded by MDV, we used RT-qPCR analysis to compare the relative expression levels of the MDV-1 genes pp38, gB, meq, RLORF6, UL6, UL13, UL42, and UL52 in CEF cells infected with the GX0101 or GX0101∆pp38 viruses. As demonstrated in Figure 2b, except for the undetectable pp38 gene in GX0101∆pp38-infected CEF cells, the mRNA expression levels of all other detected viral genes were not affected by the absence of the pp38 gene in the GX0101 virus.

### 3.3. Construction of a Pool of mAbs against MDV Proteins

Using the purified cellular proteins extracted from GX0101-infected CEFs as antigens to immunize BALB/C mice, the hybridomas secreting antibodies were first established by cell fusion. The primary screening of MDV-specific positive antibodies was performed by IFA staining, and 34 hybridomas in total were identified, which secreted positive supernatants with specific reactivity to the MDV plaques produced by the infection with GX0101 in the CEF monolayers rather than the mock CEF cells. As listed in Table 3, the 34 positive hybridomas can be divided into 3 groups: the first group, containing 15 positive hybridomas, was conserved to all three serotypes of MDV; the second group, composed of 11 positive hybridomas, was reactive to both MDV-1 and MDV-3 viruses, while the third group of the remaining 8 positive hybridomas was MDV-1 specific.

### 3.4. Cross-Screening and Identification of pp38-Specific mAbs

**Figure 3:**
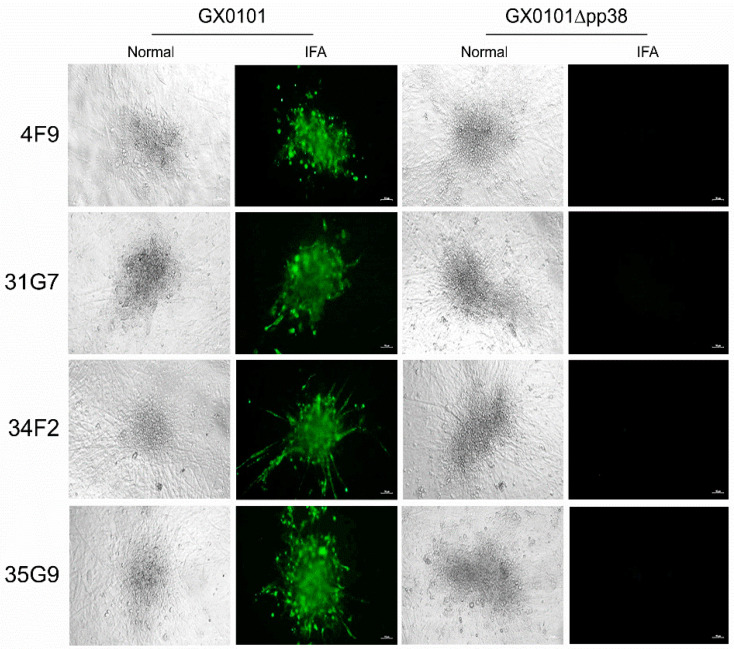
Cross-screening of pp38-specific monoclonal antibodies by immunofluorescence assay. Plaques produced by infection of CEFs with GX0101 or GX0101∆pp38 were stained with 4 different antibodies and visualized by immunofluorescence and bright-field microscopy. IFA, immunofluorescence assay; Merge, fused image. Scale bar = 50 µm.

**Figure 4:**
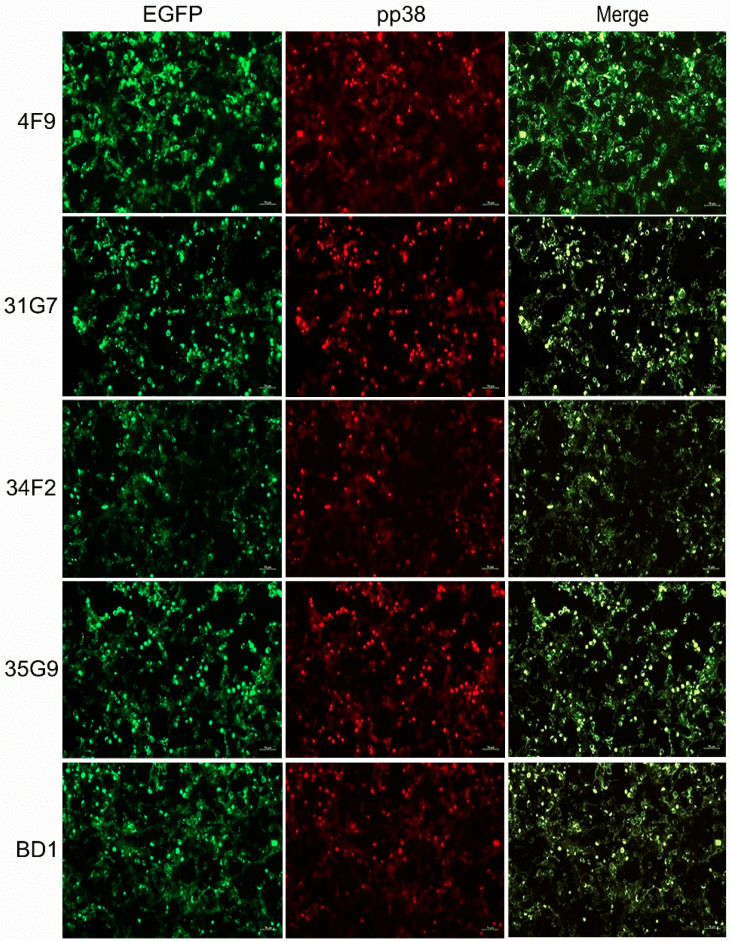
Staining of pp38 proteins overexpressed in 293T cells by immunofluorescence assay. The pp38 mAb BD1 served as a positive control. EGFP, enhanced green fluorescent protein with auto-fluorescence in green; pp38, MDV-1 phosphoprotein 38 stained in red; Merge, fused image in yellow. Scale bar = 50 µm.

**Figure 5:**
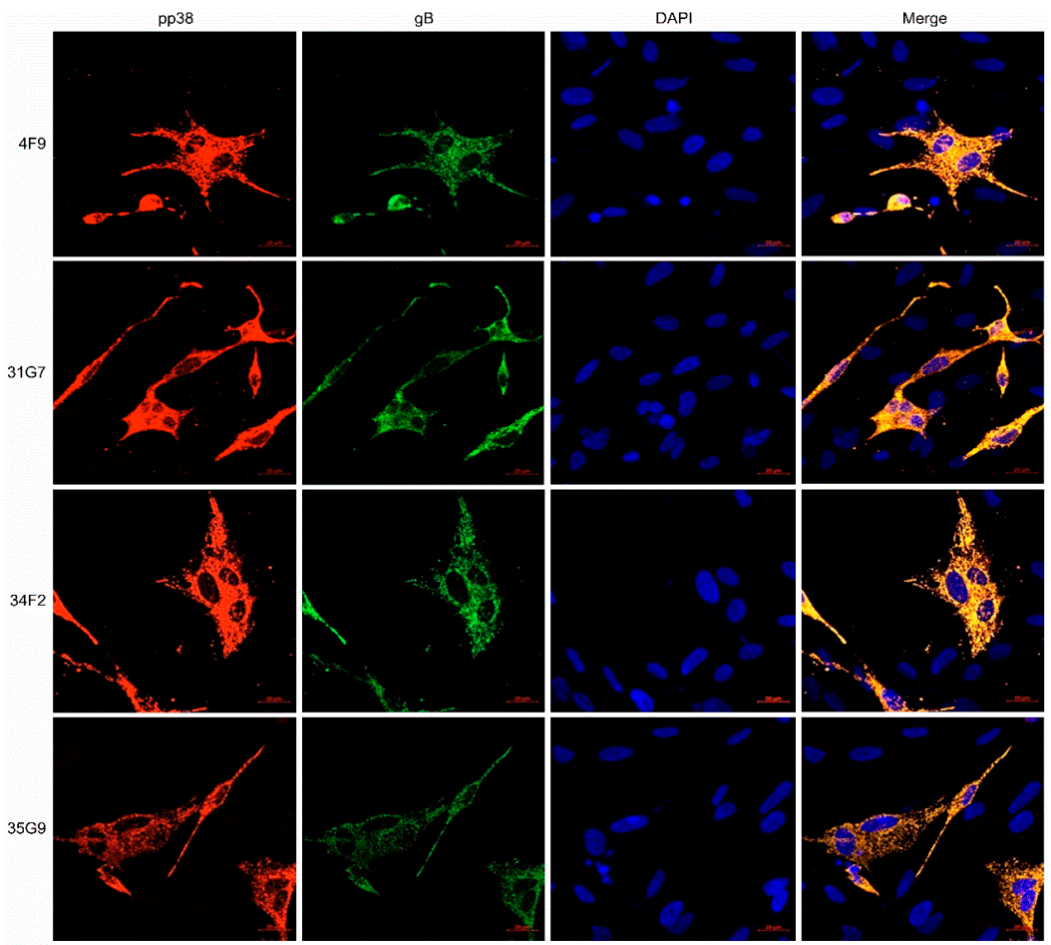
Confocal analysis of pp38 and gB proteins in MDV-infected CEFs. pp38, MDV-1 phosphoprotein 38 stained in red; gB, MDV-1 glycoprotein B stained in green; DAPI, 4′,6-diamidino-2-phenylindole used to indicate the nuclei of CEFs in blue; Merge, fused image in yellow. Scale bar = 20 µm.

For the screening and identification of pp38-specific mAbs, IFA staining was performed to discriminate all of the 34 MDV-specific antibodies using MDV plaques produced in CEF monolayers infected with parental GX0101 or the pp38-deleted mutant virus. As demonstrated in Figure 3, the supernatants of four positive hybridomas—namely 4F9, 31G7, 34F2, and 35G9—were observed to specifically react to the viral plaques formed in GX0101-infected CEFs, but not to those produced by GX0101∆pp38. Thus, the preliminary data suggested that these four antibodies were pp38-specific mAbs. For further confirmation of the specificity of these mAbs to MDV-1 pp38, 293T cells were transfected with the pEGFP-N1-pp38 plasmid to express the pp38 protein, and its reactivity to mAbs was detected by IFA. As shown in Figure 4, the IFA staining showed that the overexpressed pp38 proteins specifically stained in red by all four distinct mAbs were all identically co-localized with the spontaneous EGFP green fluorescence in 293T cells, similar to the positive control anti-pp38 mAb BD1. The data confirmed that all four mAbs could specifically recognize the MDV-1-specific pp38 proteins expressed in eukaryotic cells. Utilizing confocal experiments, we also analyzed the intracellular staining by pp38 mAbs in GX0101-infected CEF cells, which demonstrated that pp38 was stained red by all four mAbs (4F9, 31G7, 34F2, and 35G9) with a cytoplasmic distribution, coinciding with the results of mAb HB3 staining of gB proteins (Figure 5). Furthermore, ascitic fluids of hybridoma 31G7 were prepared from BALB/C mice, and were characterized and applied as detailed below. The results showed that titer of the ascitic fluids of mAb 31G7 was very high, remaining positive even up to the dilution of 1:128,000, as determined by IFA. The isotype of mAb 31G7 was characterized to be IgG1/kappa.

### 3.5. Characterization of the MDV-1 pp38-Specific mAb 31G7

**Figure 6:**
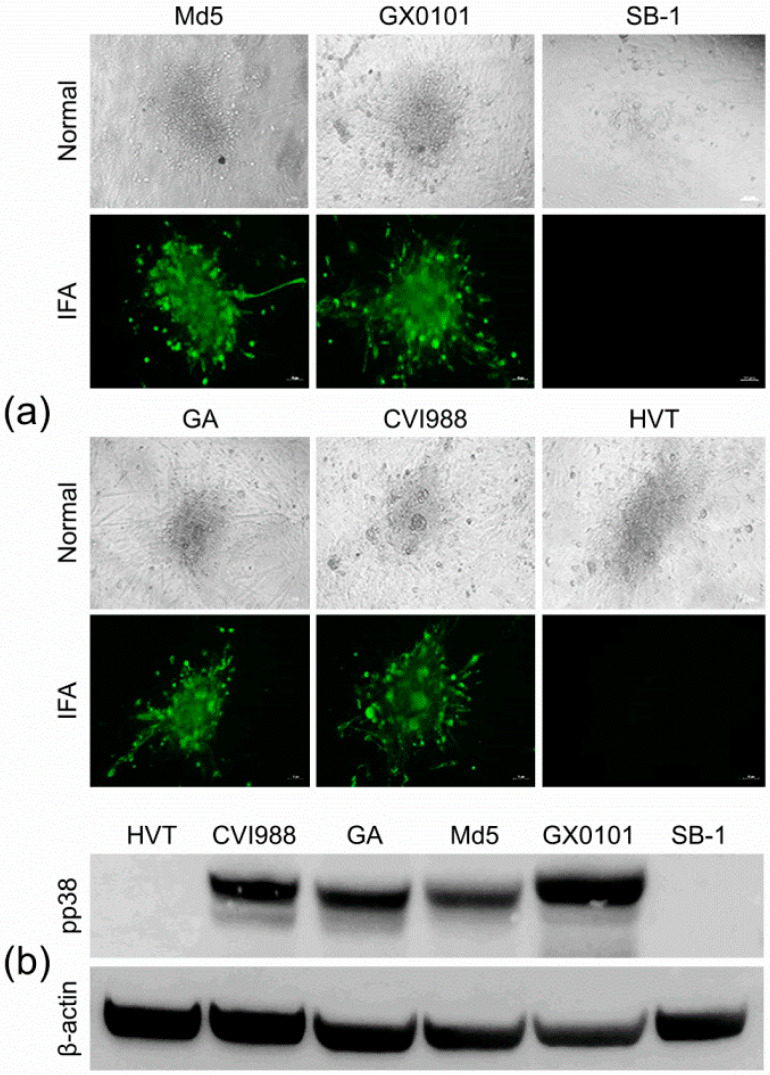
Reactivity of the mAb 31G7 to different serotypes of MDVs: (**a**) Immunofluorescence assays performed for the detection of the reaction spectrum of the pp38 mAb 31G7 to different serotypes of MDVs. Normal images of GX0101 or GX0101∆pp38 plaques under regular light; IFA, immunofluorescence assay. (**b**) Western blot analysis performed for the detection of the reaction spectrum of the mAb 31G7 to proteins from CEFs infected with different serotypes of MDVs. Chicken β-actin was used as the protein loading control. Scale bar = 50 µm.

**Figure 7:**
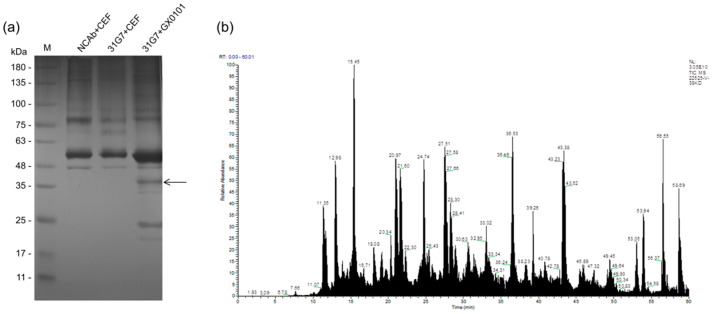
Immunoprecipitation and mass spectrometry analysis of the proteins recognized by the pp38 mAb 31G7: (**a**) Image of the immunoprecipitation and silver-stained SDS gel showing obvious protein bands captured by the pp38 mAb 31G7. The black arrow indicates the band of a target protein about 38 kD in size. (**b**) Image of total ion chromatography (TIC) for the 38 kD target band analyzed by LC–MS/MS.

**Figure 8:**
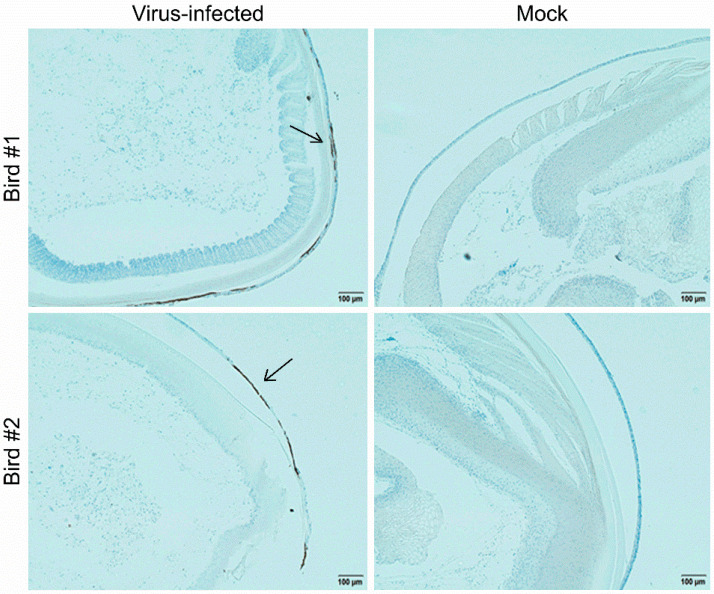
Expression of pp38 proteins in the feather follicle epithelium of MDV-infected birds, as determined by immunohistochemistry. The feather follicular samples from Md5-challenged birds at 14 days post-infection were subjected to immunohistochemistry analysis using the pp38 mAb 31G7. The black arrows indicate MDV-expressed pp38 proteins in the feather follicle epithelium. Scale bar = 100 µm.

For further characterization of the newly developed MDV-1 pp38-specific mAbs, the reaction spectra of the mAb 31G7 against all three serotypes of MDV—including virulent MDV-1 strains with different virulence, as well as vaccine strains—were determined by IFA and Western blot analysis. IFA analysis demonstrated that the viral plaques in CEF monolayers produced by infection with different MDV-1 strains—including Md5, GX0101, GA, and CVI988—were specifically stained with green fluorescence after the incubation with mAb 31G7, while the viral plaques produced by the HVT or SB-1 viruses did not show any fluorescence staining (Figure 6a). The Western blot analysis also showed similar results to those of IFA staining. As demonstrated in Figure 6b, none of specific reaction bands were observed in the samples collected from HVT- or SB-1-infected CEF cells, but in those collected from CEFs infected with MDV-1 strains a strong signal of protein bands with a molecular weight about 38 kD was observed, as expected, consistent with the molecular size of MDV-1-specific pp38 proteins. Furthermore, the mAb 31G7 was also used to perform IP with the proteins that were extracted and purified from MDV-1-infected CEFs. The results showed that bands of the mAb heavy-chain-specific proteins (56 kD) appeared in all three sample lanes of the GX0101-infected or mock control CEF cells, while in the fourth lane of proteins harvested by the mAb 31G7, several specific bands with different molecular weights were observed, including a specific band about 38 kD in size (Figure 7a). This band was purified and analyzed by the subsequent LC–MS/MS analysis, which also confirmed its identity as an MDV-1-specific pp38 protein (Figure 7b). The data of mass spectrometry analysis were shown in Tables S1 and S2. The pp38 protein is a highly expressed protein in the feather follicle epithelium (FFE) of MDV-infected birds in the early stages of disease. Thus, we finally applied the mAb 31G7 to detect MDV particles in the FFE. As demonstrated in Figure 8, the IHC analysis clearly confirmed that the mAb 31G7 was also a perfect reagent for displaying the existence of lytic MDV particles in the FFE of virus-infected birds.

## 4. Discussion

It is well known that high purity and structural features that maintain the integrity of epitopes of viral proteins are crucial for the successful generation of specific mAbs against viral proteins. For the generation of some of the early MDV-specific mAbs in the 1980s–1990s, MDV immunogens were prepared by homogenization or sonication of repeatedly frozen and thawed virus-infected CEF cultures [9,10,11]. Although several MDV-specific hybridomas were developed, the identities of many of these remained unknown due to the limitations in the experimental technology. In a number of studies where specific mAbs against MDV proteins such as gB, gD, gp82, pp38, ICP4, and ORF873 were developed [22,23,24,25,26,27], recombinant proteins expressed in baculovirus systems were used as immunogens. MDV encodes more than 100 viral proteins or hypothetical candidates. However, to date, only a few MDV-specific mAbs with clear identities—such as MEQ, gC, gH, UL46 to UL49 VP5, VP11/12, VP13/14, UL48 (VP16), UL49 (VP22), RR1, and RR2 [28,29,30,31,32,33,34,35]—have been developed in recent years. Monoclonal antibodies against most MDV proteins are ideally needed to meet the research needs for revealing MDV’s pathogenesis/oncogenesis and the development of MD diagnostic reagents. To establish an antibody pool against as many MDV proteins as possible, we used the NE-PER^®^ Nuclear and Cytoplasmic Extraction Reagents to extract both the cytoplasmic and nuclear proteins from the MDV-infected CEF cells, without changing the original structural features of the viral proteins, to make antigens for the immunization of mice for the production of hybridomas. Through the rapid screening method of IFA staining, a pool of MDV antibodies containing 34 hybridomas was successfully established, containing an abundance of mAbs conserved to all three serotypes of MDVs, or specific only to MDV-1. As we expected, this provides an important basis for the subsequent identification of mAbs against specific viral proteins of MDV.

For future applications, a new efficient strategy must first be developed for the rapid and accurate identification of specific viral protein mAbs from an antibody pool. As a new generation of gene-editing technology, CRISPR/Cas9 can specifically, efficiently, and almost omnipotently modify the target genomic sites or gene sequences of animals, plants, and even viruses [12]. Recently, MDV has also been successfully reconstituted by the CRISPR/Cas9 system to generate gene-knockout or recombinant viruses with foreign sequences inserted for the study of viral gene functions and/or the development of recombinant vaccines [13,14,15,16,17,18,19,20]. In the present study, we successfully generated the pp38-deleted MDV mutant GX0101∆pp38 utilizing the CRISPR/Cas9-based gene-editing technology, and first applied this mutant in the cross-screening and identification of MDV-1 pp38-specific mAbs, together with its parental virus. From the antibody pool of 34 hybridomas secreting MDV-specific antibodies, four pp38-specific mAbs were easily picked up and identified with clear features to specifically recognize the pp38 proteins and/or MDV particles in virus-infected CEFs. These were further verified by IFA staining, Western blotting, IP/MS, confocal microscopy, and IHC analysis. Our data indicated that the new strategy based on the CRISPR/Cas9 gene-editing technology was successfully developed for the rapid, efficient, and accurate screening and identification of mAbs against MDV-1-specific proteins, providing a meaningful reference for other DNA viruses—especially for herpesviruses.

MD is the first case in human history where cancers could be prevented by vaccination. In the past several decades, three serotypes of MDV vaccine strains—attenuated MDV-1, nonpathogenic MDV-2, and HVT—have been developed and used for the control of MD, which has immensely benefitted the poultry industry worldwide. MDV is a strictly cell-bound herpesvirus that can maintain a lifelong persistent infection in chicken hosts following infection or vaccination. Therefore, it is usually not easy to quickly and efficiently distinguish the epidemic virulent MDV infection from vaccinations for clinical diagnosis. The protein pp38 is a recognized MDV-1-specific protein that is highly expressed in visceral parenchymal tumor tissues and MDV-transformed lymphoblastoid cell lines [36]. Thus it is an excellent diagnostic marker not only for the differentiation of MDV infection and vaccination, but also for distinguishing clinical cases of the major avian neoplastic diseases with similar clinical symptoms and visceral tumors, such as MD, avian leukemia (AL), and reticuloendotheliosis (RE). As one of the important pathogenic features of MDV-1, pp38 is expressed earlier and at higher levels in virus-infected birds. Therefore, the development of mAbs against MDV-1-specific pp38 protein can provide key reagents for future usage in the differential diagnosis of MDV infection and vaccination. Previously, only researchers from the United States [10], Japan [37], and the United Kingdom [38] have reported the generation of pp38-specific mAbs. Indeed, the pp38 mAb H19 has been found to be useful for distinguishing the MDV-1 vaccine strain CVI988 from epidemic strains with varying virulence [39], further supporting the notion that the pp38 protein can be used as an ideal diagnostic marker. In this study, although the four pp38-specific mAbs we developed did not display the ability to distinguish MDV-1 vaccine strains from epidemic strains, the mAb 31G7 demonstrated a wide reaction spectrum, and was usable for all of the immunoassays that we tested, such as IFA, confocal microscopy, Western blotting, IP, and IHC analysis. Whether these pp38 mAbs can be used for the differential diagnosis of MD and other avian neoplastic diseases merits further investigation. It is undoubted that with the newly developed CRISPR/Cas9-based strategy for the efficient identification of mAbs, more useful antibodies against virus-specific proteins can be easily produced for future virology research.

## Figures and Tables

**Table 1:** Primers used for quantitative RT-qPCR analysis.

Primer Pair	Primer Name	Sequence (5′-3′)
1	pp38-F	CCGAAAGACAAAACCCAAAT
	pp38-R	ATGTAACCAGCATATAAGAACGC
2	gB-F	TCTAGGGCATGGCACACGAC
	gB-R	GAATACGGAAACACAGAGCGG
3	meq-F	AGCCGGAGAGGCTTTATGC
	meq-R	GGCCCGAATACAAGGAATCC
4	LORF6-F	AATGCGGATCATCAGGGTCTC
	LORF6-R	GAGAGGCTTTATGCTCGTCTTACC
5	UL6-F	GAATTCGATGTCGGCAGTAAGC
	UL6-R	TACATTCCCCACGCTCACCAC
6	UL13-F	ACCCTCGGTGACGTTAACAAAG
	UL13-R	TTCATGGATCTCGGCAAAGC
7	UL42-F	TTCGTCAGCCCTCATCGTG
	UL42-R	AAATGCGTTAGTATCTTCCAGTGC
8	UL52-F	AATGGGTTATCTCTGAAGGGTCG
	UL52-R	AGTCAGACCTCGTTTACCCCTTG

**Table 2:** Oligos and primers used for making gRNA plasmids or PCR identification of pp38-deleted MDV mutants.

gRNA/Target	Oligo/Primer	Sequence (5′-3′) *
gRF1	gRF1–5p	CACCGCGTGCATGTCATTTTTCGC
	gRF1–3p	AAACGCGAAAAATGACATGCACGC
gRF2	gRF2–5p	CACCGGTGCAGGCATTGTCGTTGG
	gRF2–3p	AAACCCAACGACAATGCCTGCACC
gRF3	gRF3–5p	CACCGGGTATGTTAGTCGGTAGAA
	gRF3–3p	AAACTTCTACCGACTAACATACCC
gRR1	gRR1–5p	CACCGAACAGAAGCGGAATGCGCCG
	gRR1–3p	AAACCGGCGCATTCCGCTTCTGTTC
gRR2	gRR2–5p	CACCGTCGCCGACGAGGCAGGGCAT
	gRR2–3p	AAACATGCCCTGCCTCGTCGGCGAC
gRR3	gRR3–5p	CACCGCGAAGGGCTGACGGCGTCTT
	gRR3–3p	AAACAAGACGCCGTCAGCCCTTCGC
pp38	pp38F	TGGTGGGGAGATAGTCTCGG
	pp38R	TGATCGGTGGTGTAACCGTG

* The flanking restriction sites of *Bbs*I were shown in red, and the extra G or C added at the 5′ (or 3′) end of each gRNA oligo were shown in blue.

**Table 3:** List of the hybridomas secreting MDV-specific monoclonal antibodies.

No.	Hybridomas	Specificity		
		MDV-1/GX0101	MDV-3/HVT	MDV-2/SB-1
1	4B1	+	+	+
2	4B5	+	+	+
3	4B8	+	+	+
4	4F11	+	+	+
5	5D5	+	+	+
6	5D10	+	+	+
7	5E8	+	+	+
8	5E10	+	+	+
9	5F6	+	+	+
10	6B1	+	+	+
11	6E4	+	+	+
12	6G9	+	+	+
13	6G11	+	+	+
14	10B2	+	+	+
15	27H3	+	+	+
16	1F5	+	+	-
17	2E2	+	+	-
18	2F3	+	+	-
19	2G4	+	+	-
20	2G6	+	+	-
21	3B7	+	+	-
22	3G3	+	+	-
23	4B5	+	+	-
24	4E7	+	+	-
25	4E11	+	+	-
26	6C4	+	+	-
27	4F9	+	-	-
28	10B1	+	-	-
29	10G9	+	-	-
30	10F9	+	-	-
31	21G9	+	-	-
32	31G7	+	-	-
33	34F2	+	-	-
34	35G9	+	-	-

“+”, indicating a positive antigen–antibody reaction; “-”, meaning a negative antigen–antibody reaction.

## Data Availability

Not applicable.

## Supplementary Materials

The following supporting information can be downloaded at: https://www.mdpi.com/article/10.3390/v14092045/s1, Table S1: Statistics of the number of peptides identified by MS_22525-V-38KD-pep; Table S2: Statistics of the number of proteins identified by MS_22525-V-38KD-protein.
